# Dexamethasone in Patients with Glioblastoma: A Systematic Review and Meta-Analysis

**DOI:** 10.3390/cancers16071393

**Published:** 2024-04-01

**Authors:** Pierre Scheffler, Christian Fung, Shahan Momjian, Dominik Koessinger, Levin Häni, Nicolas Neidert, Jakob Straehle, Florian Volz, Oliver Schnell, Jürgen Beck, Amir El Rahal

**Affiliations:** 1 Department of Neurosurgery, Medical Center University of Freiburg, 79098 Freiburg, Germany; pierre.scheffler@uniklinik-freiburg.de (P.S.);; 2 Department of Neurosurgery, Geneva University Hospital, Faculty of Medicine of Geneva, 1205 Geneva, Switzerland; 3 Department of Neurosurgery, Inselspital, Bern University Hospital, University of Bern, 3010 Bern, Switzerland; 4 Berta-Ottenstein Programme, Faculty of Medicine, University of Freiburg, 79098 Freiburg, Germany

**Keywords:** dexamethasone, dosing, glioblastoma, complications, evidence-based

## Abstract

**Simple Summary:**

Dexamethasone is frequently administered in brain tumor patients for symptomatic relief. However, an increasing number of publications suggests that dexamethasone may lead to worse outcome in patients with glioblastoma. Our study reviews all the published evidence and aggregates the available data in a meta-analysis. We found that dexamethasone indeed significantly reduces overall and progression-free survival in glioblastoma patients, even when accounting for clinical status. Given the potential detrimental association of dexamethasone use on overall survival, its administration to glioblastoma patients should be approached with caution.

**Abstract:**

Objective: Glioblastomas are the most common primary central nervous system (CNS) tumors. Although modern management strategies have modestly improved overall survival, the prognosis remains dismal, with treatment side effects often impinging on the clinical course. Glioblastomas cause neurological dysfunction by infiltrating CNS tissue and via perifocal oedema formation. The administration of steroids such as dexamethasone is thought to alleviate symptoms by reducing oedema. However, despite its widespread use, the evidence for the administration of dexamethasone is limited and conflicting. Therefore, we aimed to review the current evidence concerning the use and outcomes of dexamethasone in patients with glioblastoma. Methods: We performed a systematic review and meta-analysis according to the PRISMA-P guidelines. We performed a restricted search using the keywords “Dexamethasone” and “Glioblastoma” on PubMed, Web of Science, Cochrane Library, and Academic Search Premier. We included studies reporting on overall survival (OS) and progression-free survival (PFS) in glioblastoma patients receiving higher or lower dexamethasone doses. The risk of bias was assessed using ROBINS-I. We performed a meta-analysis using a random effects model for OS and PFS. Results: Twenty-two retrospective studies were included. Higher doses of dexamethasone were associated with poorer OS (hazard ratio 1.62, confidence interval 1.40–1.88) and PFS (1.49, 1.23–1.81). OS remained worse even when studies corrected for clinical status (1.52, 1.38–1.67). Conclusion: Despite the widespread use of dexamethasone in glioblastoma patients, its use is correlated with worse long-term outcomes. Consequently, Dexamethasone administration should be restricted to selected symptomatic patients. Future prospective studies are crucial to confirm these findings.

## 1. Introduction

Glioblastoma is the most common primary malignant tumor of the central nervous system (CNS), accounting for 48.6% of tumors, with an estimated incidence of 3.23 per 100,000 persons per year [1]. According to the current WHO classification, glioblastomas are among the most aggressive primary CNS tumors [2]. However, modern treatment strategies have improved the prognosis, with a clinical course often impinged by treatment side effects and cognitive decline. Standard treatment of glioblastomas consists of maximum safe resection followed by adjuvant chemotherapy and radiotherapy [3]. As a result, the survival rate for glioblastoma patients improved from 3.3 months to a median of 15 months in the past 30 years [4]. Most glioblastomas show rapid growth, with diffuse infiltration into healthy CNS tissue [5]. This growth is frequently associated with perifocal oedema. Although, due to necrosis, neurological function within the center of the tumor is usually irreversibly lost, neurological activity within the surrounding edematous tissue can be temporarily alleviated by reducing oedema.

Dexamethasone is a synthetic glucocorticoid that was first described in 1958 [6]. Dexamethasone is known for its high glucocorticoid potency, weak mineralocorticoid effects, and long biological half-life. The potent effects of dexamethasone administration on symptoms in brain tumor patients were first described by Galicich et al. [7,8]. Since then, dexamethasone has been widely used in patients with intracranial tumors. Steroids, like dexamethasone, are believed to mitigate the symptoms experienced by intracranial tumor patients owing to their anti-inflammatory properties. These properties facilitate the reduction of perifocal edema and potentially enhance the integrity of the blood–brain barrier. They achieve this through various mechanisms, including acting on the endothelium and exerting a direct influence on the astrocytes surrounding the vessels, consequently diminishing vasogenic perifocal edema. Administration of glucocorticoids may also improve general neurological function by reducing intracranial pressure [9]. Palombi et al. showed that administration of dexamethasone in glioblastoma patients leads to symptomatic improvements, such as a significant reduction in headaches, vomiting, seizures, and focal neurological deficits [10]. Villani et al. demonstrated a 20-point improvement in the Karnofsky Performance Score for 43.8% of patients with grade II–IV gliomas receiving dexamethasone [11].

Nonetheless, glucocorticoids are a class of drugs that affect various organ systems with unwanted side effects. Short-term glucocorticoid administration is generally deemed safe in most patients, even when given in substantial doses, such as in patients with acute worsening of multiple sclerosis [12]. However, long-term administration of high doses of glucocorticoids causes Cushing’s syndrome, which is associated with elevated cardiovascular risk, osteoporosis, and an elevated infection risk due to immunosuppression [13,14].

Although dexamethasone is widely used in patients with cerebral edema, it may not always be beneficial. Administration of dexamethasone was widespread in patients with traumatic brain injury until studies showed that its use was detrimental to outcomes [15]. More specifically, for glioblastoma patients, the use of dexamethasone has raised concerns. Recent findings suggest a potential negative impact on both PFS and OS, pointing towards a correlation with poorer long-term prognoses, making the routine use of this drug in this patient group a subject of careful consideration [16,17,18,19]. As a result, we decided to compile the currently available evidence on the impact of dexamethasone use in glioblastoma patients in a quantitative review.

## 2. Materials and Methods

### 2.1. Search Strategy

We performed a systematic review and meta-analysis according to the PRISMA-P guidelines. This review was not prospectively registered. In our meta-analysis, we included studies reporting on the objective outcomes of patients with glioblastomas taking dexamethasone, specifically their overall survival (OS) and progression-free survival (PFS). For our literature review, we searched the following databases: PubMed, Web of Science, Cochrane Library, and Academic Search Premier. All the results from all the databases were included up to 1 September 2023. We searched for all publications that included the keywords “dexamethasone” and “glioblastoma”.

### 2.2. Screening

Two independent reviewers (PS and AER) screened all the abstracts. For the qualitative discussion of the current evidence, we filtered all the publications that reported on the effects of dexamethasone in glioblastoma patients, in glioblastoma animal models, or on glioblastoma cells. For the quantitative part of the review, we selected all the publications that reported on the overall or progression-free survival in patient groups with higher and lower dexamethasone intake and those that provided data that could be used to extract hazard ratios. For studies reporting on the same patient population, the most informative study (either the most recent or the one with the largest population) was selected.

### 2.3. Data Collection

Data were collected from published reports by one reviewer (PS) and subsequently confirmed by a second reviewer (AER). Data on OS and PFS were extracted for all available studies. Data from the multivariable analysis were extracted from studies that provided both univariable and multivariable analyses. We also extracted all the variables that were part of the multivariable analysis. For Hagan et al. [20], we extracted the data from the model adjusted for preoperative blood glucose. The bias of individual studies was assessed using ROBINS-I [21] by two independent reviewers (PS and AER).

### 2.4. Analysis

We analyzed the OS and PFS based on the reported hazard ratios (HR) and confidence intervals (CI). Most of the publications reported the effect sizes and variances of the OS and PFS as hazard ratios with a corresponding confidence interval. In one case, the confidence interval was calculated based on a reported standard error. If the publications only reported Kaplan Meier plots, the HR and CI were calculated based on data extracted using Engauge Digitizer. Statistical analysis was performed using the R programming language and its metafor package. A random effects model was used to calculate the average effect size across studies. Separate meta-analyses were performed for all studies reporting OS, all studies reporting PFS, and all studies reporting OS that adjusted for clinical status (by group matching or considering the Karnofsky performance score or ECOG performance status in their multivariable analysis), respectively. Reporting bias was assessed using funnel plots. Plots were created using the funnel function from R’s metafor package. Certainty was assessed according to the GRADE working group’s criteria [22].

## 3. Results

### 3.1. Study Selection

**Figure 1:**
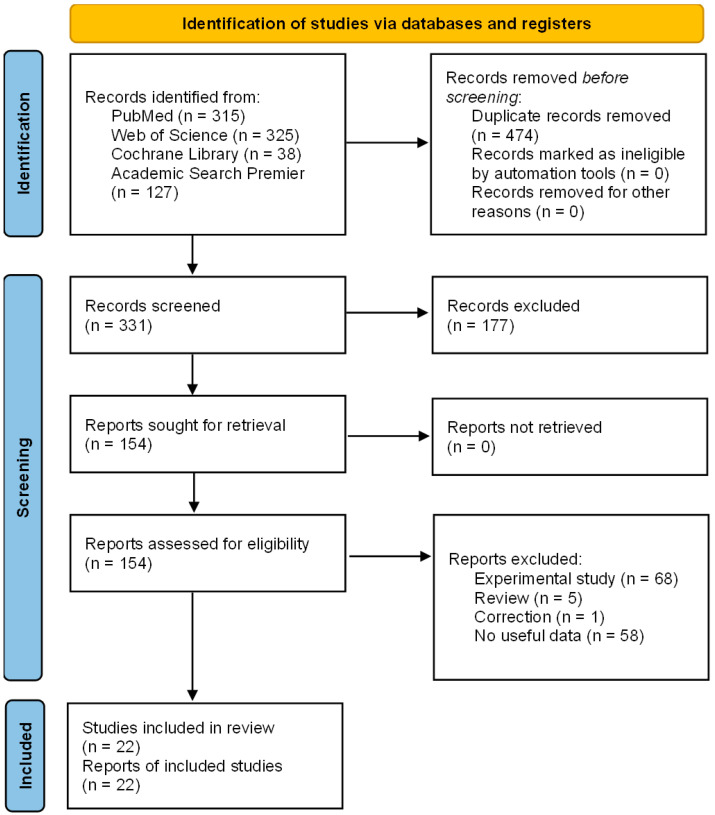
PRISMA-P flowchart detailing the study selection process. Of the 331 studies quantifying OS and PFS, 154 were retrieved for full review. After the exclusion of ineligible studies, 22 studies remained for the quantitative review.

Our literature search initially revealed 318 studies quantifying overall and progression-free survival. After screening all the abstracts, 153 studies were retrieved for full review. After the exclusion of ineligible studies, 22 studies were identified for the quantitative review [17,18,20,23,24,25,26,27,28,29,30,31,32,33,34,35,36,37,38,39,40] (Figure 1).

### 3.2. Study Characteristics

All the studies selected for quantitative review were retrospective analyses of glioblastoma patients receiving different doses of dexamethasone. The characteristics and the quality assessment of these studies are shown in Table 1.

### 3.3. Risk of Bias in Studies

The risk of bias was determined to be either moderate or serious for all the included studies Table 1. This is mainly because all the included studies were retrospective, with only about half of them adjusting the clinical performance via multivariable analyses or matching despite clinical status being considered a significant confounder for OS and PFS.

### 3.4. Results for Individual Studies

**Figure 2:**
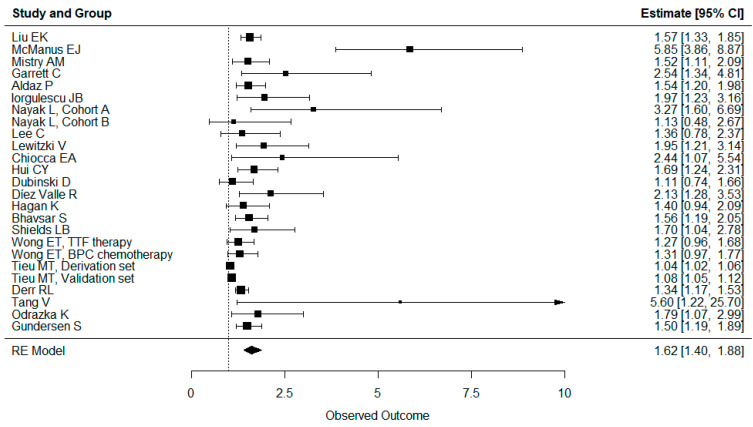
Forest plot showing all studies reporting overall survival. Administration of dexamethasone had a significant negative impact on OS (hazard ratio 1.62, confidence interval 1.40–1.88) No studies showed a positive effect of dexamethasone intake on OS or PFS.

**Figure 3:**
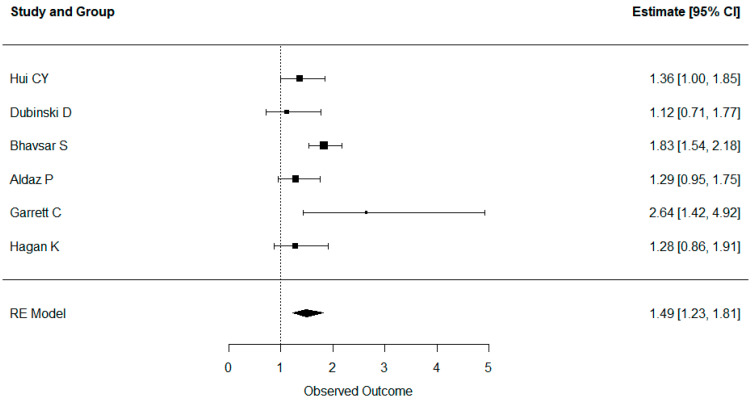
Forest plot showing all studies reporting progression-free survival. Administration of dexamethasone was correlated with worse PFS (HR 1.49, CI 1.23–1.81) in glioblastoma patients.

The hazard ratios and confidence intervals for all the studies are reported in Figure 2 and Figure 3. All the studies reported either no effect or a significantly negative effect of dexamethasone intake on both overall and progression-free survival. No studies showed a positive effect of dexamethasone intake on OS or PFS. Mistry et al. also reported that higher doses of dexamethasone had a worse effect than medium or low doses [36].

### 3.5. Results of Syntheses

The results for aggregate OS and PFS are shown in Figure 2 and Figure 3. Administration of dexamethasone had a possible significant negative impact on both OS (hazard ratio 1.62, confidence interval 1.40–1.88) and PFS (HR 1.49, CI 1.23–1.81) in glioblastoma patients. In addition, overall survival remained significantly worse, even when the studies corrected for clinical status.

**Figure 4:**
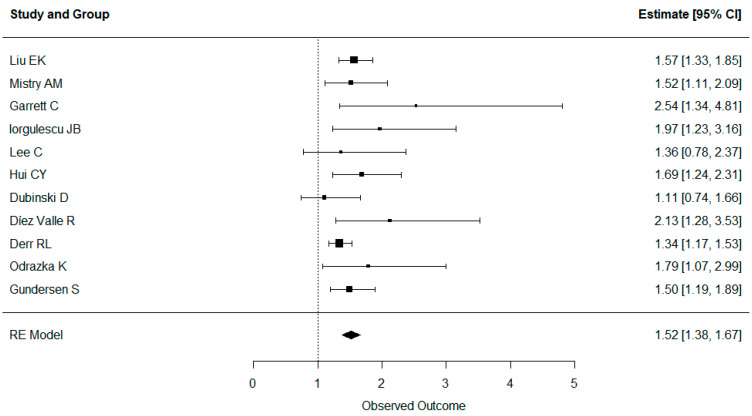
Forest plot showing all studies reporting overall survival that corrected for clinical status, as determined by the Karnofsky performance score or ECOG.

As we aimed to interrogate whether clinical status is a major confounder for survival in glioblastoma patients receiving dexamethasone, we also performed an aggregate analysis for overall survival, including only studies accounting for either the Karnofsky performance score or ECOG through matching or multivariable analysis. The results are shown in Figure 4. Even after accounting for clinical performance, dexamethasone intake had a negative impact on overall survival (HR 1.52, CI 1.38–1.67).

### 3.6. Reporting Biases

**Figure 5:**
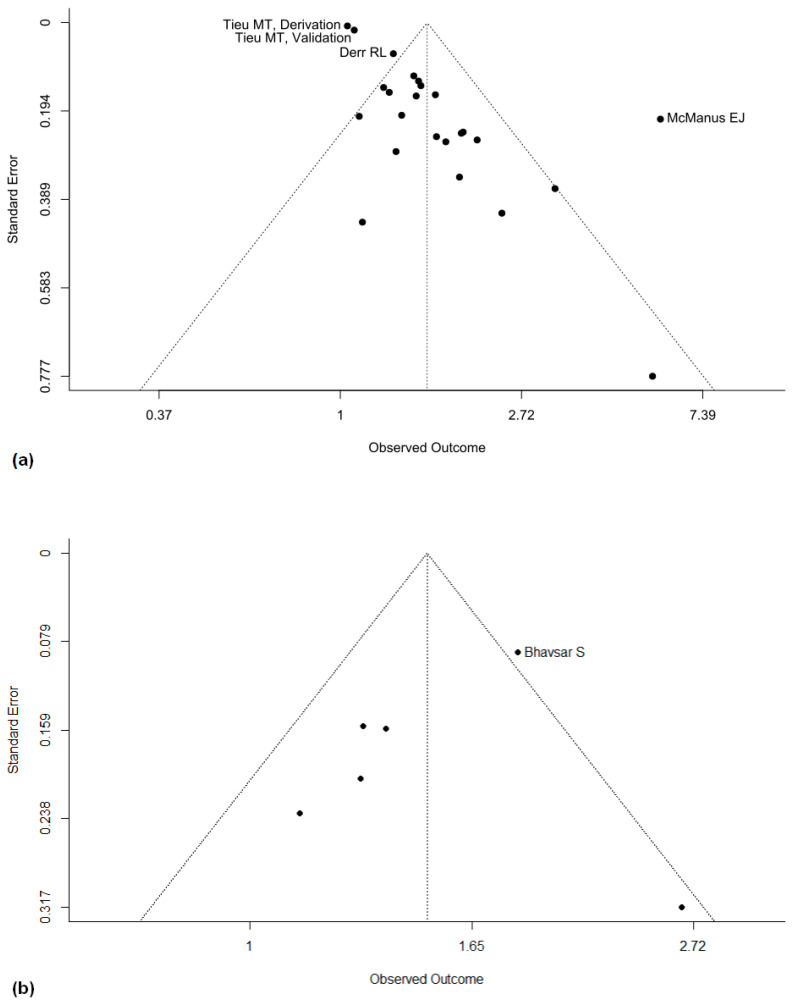
Funnel plots for all studies reporting on overall survival (**a**) and progression-free survival (**b**).

To assess the risk of bias in our study selection, we compared the study results using a funnel plot for OS and PFS (Figure 5a,b). Despite the funnel plots showing some outliers, no systematic bias could be visually perceived in the plots.

### 3.7. Certainty of Evidence

Due to the retrospective observational nature and limited patient population of all the included studies, the overall certainty of evidence for the effect of dexamethasone on OS and PFS in patients with glioblastomas according to the GRADE criteria is low.

## 4. Discussion

Studies have shown that dexamethasone reduces perifocal edema in glioblastoma patients [7] and dexamethasone significantly reduces symptoms in glioblastoma patients [10,11]. Despite the widespread use of dexamethasone in glioblastoma patients, the association of dexamethasone with overall and progression-free survival is overwhelmingly negative. There are several potential explanations for this effect. First and foremost, glucocorticoids may interfere with any immunomodulatory treatments [31,41], as well as radiation and chemotherapy [18,23,29,32,42]. In addition, dexamethasone and other glucocorticoids are immunosuppressors. As a result, the administration of dexamethasone can lead to increased infectious complications [14,43].

Glucocorticoids also lead to impaired glucose tolerance. Hyperglycemia has been shown to be an independent risk factor for overall and progression-free survival in glioblastoma patients [17,25]. Although the exact mechanisms still remain to be elucidated, one possible explanation is the Warburg effect, which proposes that tumors meet their energy needs mainly through anaerobic glycolysis [44]. In vitro, unlike human astrocytes, glioblastoma cells were shown to undergo apoptosis upon glucose withdrawal [45]. Other detrimental effects of hyperglycemia, such as an increased risk of infection, may also contribute to its negative impact on survival [46].

Preclinical studies have investigated possible mechanisms through which dexamethasone might impair survival in glioblastoma patients. Experimental models have shown that dexamethasone increases invasiveness, tumor proliferation, and angiogenesis [47]. Dexamethasone also reduces temozolomide-mediated apoptosis in human glioblastoma cells [48,49,50], possibly through O6-methylguanine-DNA methyltransferase (MGMT) upregulation, which results in an increased temozolomide resistance [51,52]. Interestingly, some preclinical studies have found that dexamethasone can also reduce glioblastoma invasiveness through various mechanisms [53,54,55,56,57].

While evidence is limited, studies indicate that the overall dose of dexamethasone applied matters. Mistry et al. showed that the higher the dose of dexamethasone received, the worse the survival, with a continuously increasing hazard ratio at cumulative doses between 30 and 512 mg within the first 3 postoperative weeks after glioblastoma resection. Patients with a cumulative postoperative dose below 75 mg had a median survival of 441 days, whereas patients who received more than 300 mg had a median survival of 183 days [36]. This dose-dependent effect may also explain the outliers we see in the funnel plots of our meta-analysis. The included studies use various cutoffs between groups with high and low dexamethasone dosing, resulting in a variety of hazard ratios even in studies with a large patient population. We aimed to account for this by using a random effects model for our analysis. Our model accounts for the large variations in dexamethasone dosing that occur in everyday clinical practice. Therefore, the aggregate hazard ratio is harder to interpret but still supports the conclusion that the administration of dexamethasone might reduce survival.

Our findings may also be affected by several different types of bias. The negative effect of dexamethasone that we show could be exacerbated by publication bias. Although the funnel plot analysis shows no significant skew, its interpretability is reduced due to the significant differences between individual study protocols. Therefore, the contribution of publication bias to our results cannot be definitely excluded. Our analysis may also be affected by confounding variables. One possible explanation for the detrimental effect of dexamethasone is that patients with worse clinical statuses and more advanced diseases with worse outcomes will receive higher doses of dexamethasone. Most guidelines suggest only administering dexamethasone in symptomatic patients and adjusting the dose according to the patient’s symptoms, and a stable or increasing corticosteroid dose is necessary for defining progressive disease in gliomas according to RANO criteria in many cases [58]. Ideally, a prospective randomized trial could determine the effect of dexamethasone on survival in glioblastoma patients. One such review has already been launched and should be completed by 2026 under the “Restrictive Use of Dexamethasone in Glioblastoma (RESDEX)” project. However, retrospective studies have already tried to account for symptom differences through multivariable analyses. Our aggregate analysis of these studies showed that dexamethasone still negatively impacts survival, independent of patient’s symptoms. Mistry et al. also used matched control groups for the Karnofsky performance score and other potential confounders such as tumor size [36]. Despite matched groups, the administration of dexamethasone still significantly decreased OS and PFS.

Given the potential detrimental association of dexamethasone use on survival, careful consideration must be given before administering dexamethasone to glioblastoma patients. As the only proven benefit of dexamethasone is the reduction of symptoms, we believe that dexamethasone should not be administered in asymptomatic patients or with a restrictive regimen. Even in symptomatic patients, alternative specific treatments may be similarly effective without affecting survival. Headaches and nausea may be treated with analgesics and antiemetics. Several non-steroidal drugs and drug combinations are also being investigated as possible edema-reducing, glucocorticoid-sparing agents, such as bevacizumab [59] and a combination of spironolactone, ecallantide, and clotrimazole [60]. However, one should consider that the use of drugs like bevacizumab is constrained due to potential complications related to wound healing. Specifically, its administration must be discontinued at least 28 days pre- and post-surgery, which can limit its daily use [61,62]. As for the postoperative period, the current high costs associated with bevacizumab may render it limited for routine use in managing symptomatic patients. However, this might change in the future.

## 5. Limitations

As a meta-analysis, our study is limited by the quality of the underlying studies. All the included studies were retrospective. As a result, there was a significant potential for selection bias, confounding variables, and other sources of bias that may have affected the validity of the results. Additionally, the studies included in the analysis showed considerable heterogeneity in their methods used for measuring dexamethasone exposure and their cutoffs for defining high and low dexamethasone doses, which reduces the interpretability of our results and the effect sizes in our analysis. Despite these limitations, until the publication of prospective trials, a systematic review of these publications represents the highest quality of evidence currently available concerning the use of dexamethasone in glioblastoma patients.

## 6. Conclusions

Given the potential detrimental association of dexamethasone use on overall survival, its administration to glioblastoma patients should be approached with caution. As the only proven benefit of dexamethasone is the reduction of symptoms, we believe that dexamethasone should not be administered in asymptomatic patients or with a restrictive regimen and only after carefully weighing the expected symptomatic improvement against the worsened prognosis. Nevertheless, this recommendation may be subject to change as future prospective studies emerge.

## Figures and Tables

**Table 1:** Details about all the studies included in this review. Abbreviations: PMID–PubMed ID, OS–overall survival, PFS–progression-free survival, KPS–Karnofsky performance score, IDH–IDH mutation status, MGMT–MGMT mutation status, ECOG–ECOG performance status, EOR–extent of resection, BMI–body mass index, RT–radiotherapy, ASA–ASA score, Charlson CI–Charlson comorbidity index, FMI–functional measure of independence.

First Author	Year	PMID	Groups	*n* (Patients)	Survival Data	Dexamethasone Dosing	Adjusted Variables	ROBINS-I Risk of Bias Assessment
Liu EK	2023	36382106		89	OS	Time-weighted dexamethasone dose (mg/day)	Age, KPS, MGMT, blood glucose, methylation subclass, subclass glucose interaction	Moderate
McManus EJ	2022	35096403		170	OS	More or less than 10 mg/d	None	Serious
Mistry AM	2021	34594571		360	OS	More or less than 200 mg total in first 3 postoperative weeks	Age, KPS, IDH, MGMT, temozolomide, RT, tumor volume, EOR, blood glucose	Moderate
Garrett C	2021	34138894		87	OS and PFS	Daily dexamethasone or not	None for OS, age, ECOG, gender, BMI, IDH, temozolomide, previous surgery, EOR for PFS	Moderate
Aldaz P	2021	33478100		285	OS and PFS	Dexamethasone yes or no postoperatively	None	Serious
Iorgulescu JB	2021	33239433		181	OS	Low (1 and 2.5 mg/kg/d) or high (10 mg/kg/d) doses	Age, KPS, MGMT, tumor volume, EOR	Moderate
Nayak L	2021	33199490	Cohorts A and B	80	OS	Any vs. no baseline dexamethasone use in patients with recurrent glioblastoma	None	Serious
Lee C	2020	32648384		125	OS	More or less than 2 mg/d after initiation of radiochemotharapy	Age, sex, ECOG, EOR	Moderate
Lewitzki V	2019	31831026		152	OS	Any vs. no dexamethasone during radiotherapy	MGMT, recurrence, salvage therapy	Serious
Chiocca EA	2019	31413142		31	OS	More or less than 20 mg within 2 weeks postoperatively after recurrent resection	None	Serious
Hui CY	2019	30864102		319	OS and PFS	More or less than 4 mg/d during radiochemotherapy	Age, KPS, sex, race, EOR, MGMT, RT	Moderate
Dubinski D	2018	29349612		113	OS and PFS	12 mg dexamethasone vs. none preoperatively	None	Serious
Díez Valle R	2018	29107723		131	OS	Dexamethasone yes or no at 2 months postoperatively	Age, sex, KPS, MGMT, EOR, time to surgery	Moderate
Hagan K	2016	27438798		162	OS and PFS	Dexamethasone yes or no preoperatively	Age, ASA, blood glucose	Serious
Bhavsar S	2016	27396375		841	OS and PFS	Dexamethasone yes or no perioperatively	Age, BMI, sex, ASA, Charlson CI	Moderate
Shields LB	2015	26520780		73	OS	Dexamethasone yes or no during radiotherapy	None	Serious
Wong ET	2015	26125449	TTF therapy and BPC chemotherapy	35	OS	Dexamethasone over or less than 4.1 mg/d in recurrent glioblastoma	None	Serious
Tieu MT	2015	26015297	Derivation and validation sets	359	OS	Mean TWM dexamethasone dose per mg during radiotherapy	Age, ECOG, blood glucose, BMI, EOR	Moderate
Derr RL	2009	19139429		191	OS	Mean daily dexamethasone dose per 10 mg/d	Age, KPS, blood glucose	Moderate
Tang V	2008	18500499		18	OS	More or less than 8 mg/d upon admission in rehabilitation	FMI	Serious
Odrazka K	2003	14628127		85	OS	More or less than 2 mg/d before radiotherapy	Age, ECOG, EOR	Moderate
Gundersen S	1996	9073058		495	OS	Dexamethasone yes or no on admission	KPS, age, resection	Moderate

## Data Availability

The authors will make the raw data supporting this article’s conclusions available upon request.

## References

[1] Ostrom Q.T., Cioffi G., Gittleman H., Patil N., Waite K., Kruchko C., Barnholtz-Sloan J.S. (2019). CBTRUS Statistical Report: Primary Brain and Other Central Nervous System Tumors Diagnosed in the United States in 2012–2016. Neuro-Oncology.

[2] Louis D.N., Perry A., Wesseling P., Brat D.J., Cree I.A., Figarella-Branger D., Hawkins C., Ng H.K., Pfister S.M., Reifenberger G. (2021). The 2021 WHO Classification of Tumors of the Central Nervous System: A summary. Neuro-Oncology.

[3] Stupp R., Mason W.P., van den Bent M.J., Weller M., Fisher B., Taphoorn M.J.B., Belanger K., Brandes A.A., Marosi C., Bogdahn U. (2005). Radiotherapy plus Concomitant and Adjuvant Temozolomide for Glioblastoma. N. Engl. J. Med..

[4] Delgado-López P.D., Corrales-García E.M. (2016). Survival in glioblastoma: A review on the impact of treatment modalities. Clin. Transl. Oncol..

[5] Xie Q., Mittal S., Berens M.E. (2014). Targeting adaptive glioblastoma: An overview of proliferation and invasion. Neuro-Oncology.

[6] Glen A.E., Johnston D.B.R., Fried J., Spooncer W.W., Hoff D.R., Sarett L.H. (1958). 16-Methylated Steroids. I. 16α-Methylated Analogs of Cortisone, a New Group of Anti-Inflammatory Steroids. J. Am. Chem. Soc..

[7] Galicich J.H., French L.A., Melby J.C. (1961). Use of dexamethasone in treatment of cerebral edema associated with brain tumors. J. Lancet.

[8] McClelland S., Long D.M. (2008). Genesis of the Use of Corticosteroids in the Treatment and Prevention of Brain Edema. Neurosurgery.

[9] Alberti E., Hartmann A., Schreckenberger F., Schütz H.-J. (1978). The effect of large doses of dexamethasone on the cerebrospinal fluid pressure in patients with supratentorial tumors. J. Neurol..

[10] Palombi L., Marchetti P., Salvati M., Osti M.F., Frati L., Frati A. (2018). Interventions to Reduce Neurological Symptoms in Patients with GBM Receiving Radiotherapy: From Theory to Clinical Practice. Anticancer. Res..

[11] Villani V., Pace A., Vidiri A., Tanzilli A., Sperati F., Terrenato I., Mariantonia C., Casini B., Metro G., Maschio M. (2019). Phase II study of weekly carboplatin in pretreated adult malignant gliomas. J. Neuro-Oncol..

[12] Hauser S.L., Cree B.A. (2020). Treatment of Multiple Sclerosis: A Review. Am. J. Med..

[13] Lacroix A., Feelders R.A., Stratakis C.A., Nieman L.K. (2015). Cushing’s Syndrome. Lancet.

[14] Youssef J., Novosad S.A., Winthrop K.L. (2016). Infection Risk and Safety of Corticosteroid Use. Rheum. Dis. Clin. N. Am..

[15] Alderson P., Roberts I. (2005). Corticosteroids for Acute Traumatic Brain Injury. Cochrane Database Syst. Rev..

[16] Pitter K.L., Tamagno I., Alikhanyan K., Hosni-Ahmed A., Pattwell S.S., Donnola S., Dai C., Ozawa T., Chang M., Chan T.A. (2016). Corticosteroids Compromise Survival in Glioblastoma. Brain.

[17] Tieu M.T., Lovblom L.E., McNamara M.G., Mason W., Laperriere N., Millar B.-A., Ménard C., Kiehl T.-R., Perkins B.A., Chung C. (2015). Impact of glycemia on survival of glioblastoma patients treated with radiation and temozolomide. J. Neuro-Oncol..

[18] Shields L.B.E., Shelton B.J., Shearer A.J., Chen L., Sun D.A., Parsons S., Bourne T.D., LaRocca R., Spalding A.C. (2015). Dexamethasone administration during definitive radiation and temozolomide renders a poor prognosis in a retrospective analysis of newly diagnosed glioblastoma patients. Radiat. Oncol..

[19] Zhou L., Shen Y., Huang T., Sun Y., Alolga R.N., Zhang G., Ge Y. (2021). The Prognostic Effect of Dexamethasone on Patients With Glioblastoma: A Systematic Review and Meta-Analysis. Front. Pharmacol..

[20] Hagan K., Bhavsar S., Arunkumar R., Grasu R., Dang A., Carlson R., Cowles C., Arnold B., Potylchansky Y., Rahlfs T.F. (2017). Association Between Perioperative Hyperglycemia and Survival in Patients With Glioblastoma. J. Neurosurg. Anesthesiol..

[21] Sterne J.A.C., Hernán M.A., Reeves B.C., Savović J., Berkman N.D., Viswanathan M., Henry D., Altman D.G., Ansari M.T., Boutron I. (2016). ROBINS-I: A tool for assessing risk of bias in non-randomised studies of interventions. BMJ.

[22] Guyatt G.H., Oxman A.D., Vist G.E., Kunz R., Falck-Ytter Y., Alonso-Coello P., Schünemann H.J., Grade Working Group (2008). Grade: An emerging consensus on rating quality of evidence and strength of recommendations. BMJ.

[23] Aldaz P., Auzmendi-Iriarte J., Durántez M., Lasheras-Otero I., Carrasco-Garcia E., Zelaya M.V., Bragado L., Olías-Arjona A., Egaña L., Samprón N. (2021). Identification of a Dexamethasone Mediated Radioprotection Mechanism Reveals New Therapeutic Vulnerabilities in Glioblastoma. Cancers.

[24] Bhavsar S., Hagan K., Arunkumar R., Potylchansky Y., Grasu R., Dang A., Carlson R., Cowels C., Arnold B., Rahlfs T. (2016). Preoperative statin use is not associated with improvement in survival after glioblastoma surgery. J. Clin. Neurosci..

[25] Derr R.L., Ye X., Islas M.U., Desideri S., Saudek C.D., Grossman S.A. (2009). Association Between Hyperglycemia and Survival in Patients With Newly Diagnosed Glioblastoma. J. Clin. Oncol..

[26] Dubinski D., Won S.-Y., Gessler F., Quick-Weller J., Behmanesh B., Bernatz S., Forster M.-T., Franz K., Plate K.-H., Seifert V. (2018). Dexamethasone-induced leukocytosis is associated with poor survival in newly diagnosed glioblastoma. J. Neuro-Oncol..

[27] Garrett C., Becker T.M., Lynch D., Po J., Xuan W., Scott K.F., de Souza P. (2021). Comparison of neutrophil to lymphocyte ratio and prognostic nutritional index with other clinical and molecular biomarkers for prediction of glioblastoma multiforme outcome. PLoS ONE.

[28] Gundersen S., Lote K., Hannisdal E. (1996). Prognostic Factors for Glioblastoma Multiforme: Development of a prognostic index. Acta Oncol..

[29] Schloss M.H., Freidberg S.R., Heatley G.J., Lo T.C.M. (1989). Glucocorticoid Dependency as A Prognostic Factor in Radiotherapy for Cerebral Gliomas. Acta Oncol..

[30] Hui C.Y., Rudra S., Ma S., Campian J.L., Huang J. (2019). Impact of overall corticosteroid exposure during chemoradiotherapy on lymphopenia and survival of glioblastoma patients. J. Neuro-Oncol..

[31] Iorgulescu J.B., Gokhale P.C., Speranza M.C., Eschle B.K., Poitras M.J., Wilkens M.K., Soroko K.M., Chhoeu C., Knott A., Gao Y. (2021). Concurrent Dexamethasone Limits the Clinical Benefit of Immune Checkpoint Blockade in Glioblastoma. Clin. Cancer Res..

[32] Kostopoulou O.N., Mohammad A., Bartek J., Winter J., Jung M., Stragliotto G., Söderberg-Nauclér C., Landázuri N. (2018). Glucocorticoids promote a glioma stem cell-like phenotype and resistance to chemotherapy in human glioblastoma primary cells: Biological and prognostic significance. Int. J. Cancer.

[33] Lee C., Ahn S., Park J.-S., Song J.H., Hong Y.-K., Jeun S.-S. (2020). Effect of Cumulative Dexamethasone Dose during Concomitant Chemoradiation on Lymphopenia in Patients with Newly Diagnosed Glioblastoma. Brain Tumor Res. Treat..

[34] Lewitzki V., Klement R.J., Kosmala R., Lisowski D., Flentje M., Polat B. (2019). Accelerated hyperfractionated radiochemotherapy with temozolomide is equivalent to normofractionated radiochemotherapy in a retrospective analysis of patients with glioblastoma. Radiat. Oncol..

[35] McManus E.J., Frampton C., Tan A., Phillips M.C.L. (2022). Metabolics risk factors in a New Zealand glioblastoma cohort. Neuro-Oncol. Pract..

[36] Mistry A.M., Jonathan S.V., Monsour M.A., Mobley B.C., Clark S.W., Moots P.L. (2021). Impact of postoperative dexamethasone on survival, steroid dependency, and infections in newly diagnosed glioblastoma patients. Neuro-Oncol. Pract..

[37] Nayak L., Molinaro A.M., Peters K., Clarke J.L., Jordan J.T., de Groot J., Nghiemphu L., Kaley T., Colman H., McCluskey C. (2021). Randomized Phase Ii and Biomarker Study of Pembrolizumab Plus Bevacizumab Versus Pembrolizumab Alone for Patients with Recurrent Glioblastoma. Clin. Cancer Res..

[38] Odrazka K., Petera J., Kohlova T., Dolezel M., Vaculikova M., Zouhar M., Malek V., Hobza V., Latr I., Nemecek S. (2003). Prognostic Impact of Hemoglobin Level Prior to Radiotherapy on Survival in Patients with Glioblastoma. Strahlenther. Onkol..

[39] Tang V., Rathbone M., Dorsay J.P., Jiang S., Harvey D. (2008). Rehabilitation in Primary and Metastatic Brain Tumours: Impact of Functional Outcomes on Survival. J. Neurol..

[40] Wong E.T., Lok E., Gautam S., Swanson K.D. (2015). Dexamethasone Exerts Profound Immunologic Interference on Treatment Efficacy for Recurrent Glioblastoma. Br. J. Cancer.

[41] Brummer A.B., Yang X., Ma E., Gutova M., Brown C.E., Rockne R.C. (2022). Dose-dependent thresholds of dexamethasone destabilize CAR T-cell treatment efficacy. PLOS Comput. Biol..

[42] Linder B., Schiesl A., Voss M., Rödel F., Hehlgans S., Güllülü Ö., Seifert V., Kögel D., Senft C., Dubinski D. (2021). Dexamethasone Treatment Limits Efficacy of Radiation, but Does Not Interfere With Glioma Cell Death Induced by Tumor Treating Fields. Front. Oncol..

[43] Car M., Šteblaj S., Mitrovič G., Dolenc-Stražar Z., Popović M. (2019). Corticosteroid-Induced Immunodeficiency in a Patient with Gliomatosis Cerebri: Are Corticosteroids Indicated in All Brain Tumors?. Clin. Neuropathol..

[44] Warburg O. (1956). On the Origin of Cancer Cells. Science.

[45] Jelluma N., Yang X., Stokoe D., Evan G.I., Dansen T.B., Haas-Kogan D.A. (2006). Glucose Withdrawal Induces Oxidative Stress followed by Apoptosis in Glioblastoma Cells but not in Normal Human Astrocytes. Mol. Cancer Res..

[46] Benfield T., Jensen J.S., Nordestgaard B.G. (2007). Influence of diabetes and hyperglycaemia on infectious disease hospitalisation and outcome. Diabetologia.

[47] Luedi M.M., Singh S.K., Mosley J.C., Hassan I.S.A., Hatami M., Gumin J., Andereggen L., Sulman E.P., Lang F.F., Stueber F. (2018). Dexamethasone-mediated oncogenicity in vitro and in an animal model of glioblastoma. J. Neurosurg..

[48] Sur P., Sribnick E.A., Patel S.J., Ray S.K., Banik N.L. (2005). Dexamethasone decreases temozolomide-induced apoptosis in human gliobastoma T98G cells. Glia.

[49] Das A., Banik N.L., Patel S.J., Ray S.K. (2004). Dexamethasone protected human glioblastoma U87MG cells from temozolomide induced apoptosis by maintaining Bax:Bcl-2 ratio and preventing proteolytic activities. Mol. Cancer.

[50] Das A., Banik N.L., Ray S.K. (2008). Modulatory Effects of Acetazolomide and Dexamethasone on Temozolomide Mediated Apoptosis in Human Glioblastoma T98G and U87MG Cells. Cancer Investig..

[51] Aasland D., Reich T.R., Tomicic M.T., Switzeny O.J., Kaina B., Christmann M. (2018). Repair Gene O(6) -Methylguanine-DNA Methyltransferase Is Controlled by Sp1 and up-Regulated by Glucocorticoids, but Not by Temozolomide and Radiation. J. Neurochem..

[52] Ueda S., Mineta T., Nakahara Y., Okamoto H., Shiraishi T., Tabuchi K. (2004). Induction of the DNA repair gene O6-methylguanine—DNA methyltransferase by dexamethasone in glioblastomas. J. Neurosurg..

[53] Shannon S., Vaca C., Jia D., Entersz I., Schaer A., Carcione J., Weaver M., Avidar Y., Pettit R., Nair M. (2015). Dexamethasone-Mediated Activation of Fibronectin Matrix Assembly Reduces Dispersal of Primary Human Glioblastoma Cells. PLoS ONE.

[54] Nair M., Romero J., Mahtabfar A., Meleis A.M., Foty R.A., Corbett S.A. (2018). Dexamethasone-Mediated Upregulation of Calreticulin Inhibits Primary Human Glioblastoma Dispersal Ex Vivo. Int. J. Mol. Sci..

[55] Lin Y.-M., Jan H.-J., Lee C.-C., Tao H.-Y., Shih Y.-L., Wei H.-W., Lee H.-M. (2008). Dexamethasone reduced invasiveness of human malignant glioblastoma cells through a MAPK phosphatase-1 (MKP-1) dependent mechanism. Eur. J. Pharmacol..

[56] Villeneuve J., Galarneau H., Beaudet M., Tremblay P., Chernomoretz A., Vallières L. (2008). Reduced Glioma Growth Following Dexamethasone or Anti-Angiopoietin 2 Treatment. Brain Pathol..

[57] Kaup B., Schindler I., Knüpfer H., Schlenzka A., Preiβ R., Knüpfer M. (2001). Time-dependent Inhibition of Glioblastoma Cell Proliferation by Dexamethasone. J. Neuro-Oncol..

[58] Leao D., Craig P., Godoy L., Leite C., Policeni B. (2020). Response Assessment in Neuro-Oncology Criteria for Gliomas: Practical Approach Using Conventional and Advanced Techniques. Am. J. Neuroradiol..

[59] Kaley T., Nolan C., Carver A., Omuro A. (2013). Bevacizumab for acute neurologic deterioration in patients with glioblastoma. CNS Oncol..

[60] Kast R., Burns T.C., Halatsch M.-E. (2021). Short review of SEC, a potential dexamethasone-sparing regimen for glioblastoma: Spironolactone, ecallantide, clotrimazole. Neurochirurgie.

[61] Abrams D.A., Hanson J.A., Brown J.M., Hsu F.P., Delashaw J.B., Bota D.A. (2015). Timing of Surgery and Bevacizumab Therapy in Neurosurgical Patients with Recurrent High Grade Glioma. J. Clin. Neurosci..

[62] Gordon C.R., Rojavin Y., Patel M., Zins J.E., Grana G., Kann B., Simons R., Atabek U. (2009). A Review on Bevacizumab and Surgical Wound Healing: An Important Warning to All Surgeons. Ann. Plast. Surg..

